# Hippocampal Ripples during Offline Periods Predict Human Motor Sequence Learning

**DOI:** 10.1523/JNEUROSCI.1502-25.2025

**Published:** 2025-10-14

**Authors:** Pin-Chun Chen, Jenny Stritzelberger, Katrin Walther, Hajo Hamer, Bernhard P. Staresina

**Affiliations:** ^1^Department of Experimental Psychology, University of Oxford, Oxford OX2 6GG, United Kingdom; ^2^Oxford Centre for Integrative Neuroimaging (OxCIN), Department of Psychiatry, University of Oxford, Oxford OX3 9DA, United Kingdom; ^3^Epilepsy Center, Department of Neurology, University Hospital Erlangen, Friedrich-Alexander-University Erlangen-Nürnberg (FAU), Erlangen 91054, Germany

**Keywords:** consolidation, hippocampus, iEEG, motor learning, offline periods, ripples

## Abstract

High-frequency bursts in the hippocampus, known as ripples (80–120 Hz in humans), have been shown to support episodic memory processes. However, converging recent evidence in rodent models and human neuroimaging suggests that the hippocampus may be involved in a wider range of memory domains, including motor sequence learning (MSL). Nevertheless, no direct link between hippocampal ripples and MSL has been established yet. Here, we recorded intracranial electroencephalography (iEEG) from the hippocampus in 20 epilepsy patients (11 males and 9 females) during an MSL task in which participants showed steady improvement across nine 30 s typing blocks interspersed with 30 s rest (“offline”) periods. We first demonstrated that ripple rates strongly increased during rest relative to typing blocks. Importantly, ripple rates during rest periods tracked behavioral improvements, both across learning blocks and across participants. These findings suggest that hippocampal ripples during rest periods play a role in facilitating motor sequence learning.

## Significance Statement

This study provides the first direct evidence that hippocampal ripples, brief high-frequency oscillations previously linked to episodic memory, also play a role in human motor sequence learning. By recording intracranial EEG from epilepsy patients during a motor learning task, we found that ripple rates increased during rest periods between typing blocks and closely tracked behavioral improvements in performance. These findings suggest that hippocampal ripples during offline periods may facilitate consolidation of newly acquired motor skills, extending the functional significance of ripples beyond episodic memory.

## Introduction

How does the human hippocampus contribute to learning and memory? Following the report of patient H.M. (Scoville and Milner, 1957), the distinction between hippocampus-dependent “declarative” and nonhippocampus-dependent “nondeclarative” memory has been widely accepted (Squire and Zola, 1996). However, mounting evidence has begun to challenge the exclusive role of the hippocampus in canonical forms of declarative memory (e.g., episodic memory). For instance, the hippocampus has been implicated in short-term memory (Husain, 2024), working memory (Axmacher et al., 2010), nonconscious forms of learning (Henke, 2010), as well as motor skill acquisition (Albouy et al., 2013).

Irrespective of the type of learning, another key question is at which stage of learning hippocampal contributions unfolds. That is, learning can be roughly divided into online and offline components, with the former denoting on-task acquisition and retrieval and the latter including post-acquisition rest periods, spent either awake or asleep. Interestingly, accumulating evidence points to an important role of hippocampal engagement during offline periods, even in tasks that seemingly do not require the hippocampus during acquisition (Sawangjit et al., 2018; Schapiro et al., 2019; Kim et al., 2023).

Mechanistically, hippocampal contributions to memory processes are thought to be mediated by ripples. These are brief, high-frequency oscillations (∼80–120 Hz in humans) capable of synchronizing a wide array of cortical and subcortical brain networks (Bragin et al., 1999; Buzsáki, 2015). Initially observed in rodent hippocampus, ripples during offline states have been linked to the reactivation/replay of navigational trajectories along with consolidation of spatial memories (Buzsáki and Tingley, 2018). In humans, hippocampal ripples can be reliably measured via direct recordings from patients undergoing invasive monitoring for the surgical treatment of refractory epilepsy (Bragin et al., 1999) and have been associated with memory performance during both online recall (Norman et al., 2019; Vaz et al., 2019) and offline sleep periods (Axmacher et al., 2008). Yet, the role of hippocampal ripples beyond episodic memory tasks remains unclear.

In the present study, we ask whether hippocampal ripples play a functional role in motor skill learning. To this end, we examined hippocampal ripple attributes during a motor sequence learning paradigm in epilepsy patients. We report a strong increase of ripple rates during offline rest relative to online typing periods. Importantly, these offline ripple increases were linked to learning behavior, tracking performance improvements within and across participants. These findings suggest a potential role for offline hippocampal ripples in motor sequence acquisition, motivating further investigation of their contribution to learning beyond episodic memory.

## Materials and Methods

### Participants

iEEG recordings from the human hippocampus were obtained from 20 participants (11 male; mean age, 30.5 years; range, 19–58 years; two left-handed; Table 1) undergoing invasive monitoring as part of their treatment for refractory epilepsy at the University Hospital Erlangen. Seventeen participants (8 male; mean age, 31.1 years; range, 19–58 years; two left-handed) were included in all analyses after rigorous artifact rejection (see Materials and Methods, Artifact detection and rejection). The sample size was determined based on recent human studies of similar design and methodology (Staresina et al., 2015; Ngo et al., 2020; Kunz et al., 2024). Handedness was assessed by the Edinburgh Handedness Inventory. Ethical approval was granted by the ethics commission of the Friedrich-Alexander Universität Erlangen-Nürnberg (142_12B), and written informed consent was obtained in accordance with the Declaration of Helsinki.

**Table 1.** Participant demographic and electrode information

Participant	Sex	Age	Handedness	# bipolar contacts		
				Tot.	L	R
1	M	21	R	2 (2)	2 (2)	0 (0)
2	M	20	R	0 (1)	0 (2)	0 (1)
3	M	30	R	2 (2)	0 (0)	2 (2)
4	F	20	R	2 (2)	0 (0)	2 (2)
5	F	47	R	2 (2)	2 (2)	0 (0)
6	M	39	R	2 (2)	0 (0)	2 (2)
7	F	58	L	2 (2)	1 (1)	1 (1)
8	F	23	R	2 (2)	2 (2)	0 (0)
9	M	39	R	2 (2)	0 (0)	2 (2)
10	F	36	R	2 (2)	2 (2)	0 (0)
11	M	19	L	2 (2)	0 (0)	2 (2)
12	M	23	R	0 (1)	0 (0)	0 (1)
13	F	35	R	2 (2)	0 (0)	2 (2)
14	F	24	R	2 (2)	2 (2)	0 (0)
15	F	35	R	2 (2)	2 (2)	0 (0)
16	M	26	R	2 (2)	2 (2)	0 (0)
17	F	38	R	0 (2)	0 (2)	0 (0)
18	F	29	R	2 (2)	2 (2)	0 (0)
19	M	27	R	2 (2)	2 (2)	0 (0)
20	M	21	R	2 (2)	0 (0)	2 (2)

Demographic and hippocampal contact information reported for each participant (1–20), including sex (male/female), age at time of experiment (years), total number of hippocampal contacts included in the analyses. Numbers in parentheses indicate the number of implanted hippocampal contacts prior to artifact rejection.

### Behavioral task

#### Motor sequence learning

**Figure 1.**
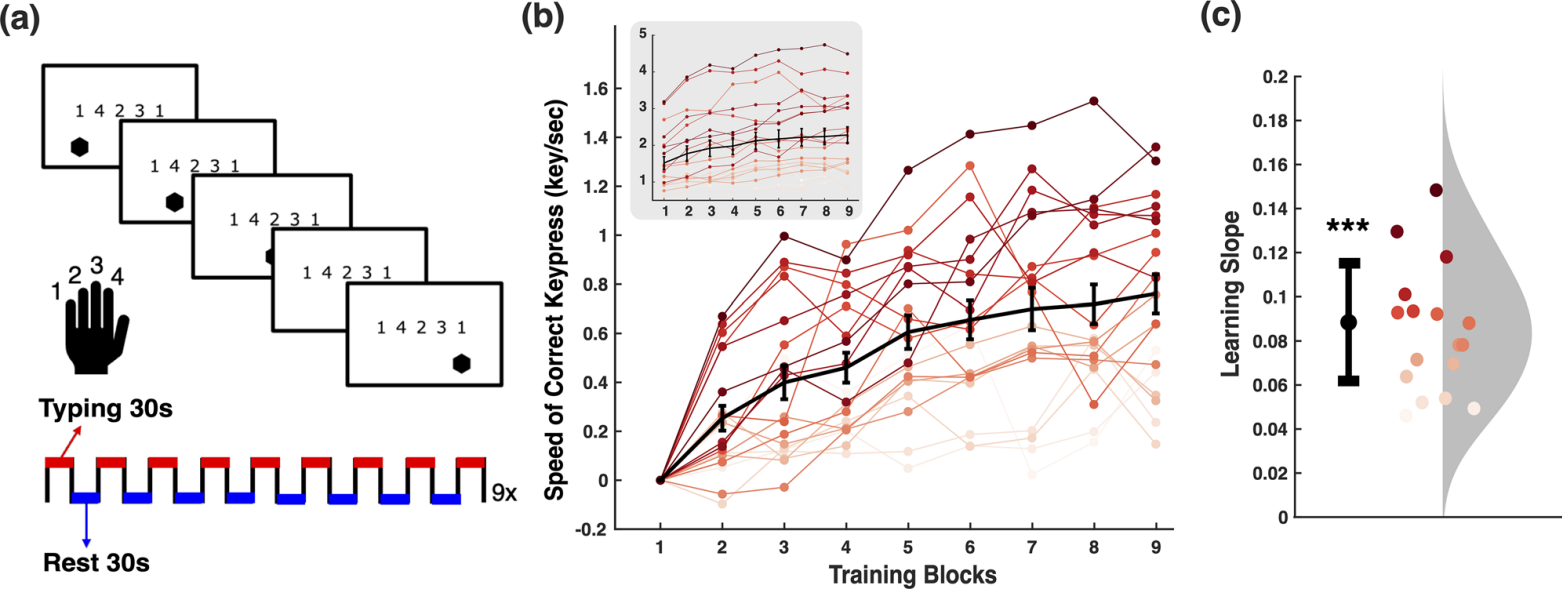
Behavioral task and performance. ***a***, Motor sequence learning (MSL) task. Participants performed an explicit MSL task with their nondominant hand where they repetitively typed a 5-item numerical sequence (1-4-2-3-1) displayed on a screen as quickly and as accurately as possible. Participants performed the typing task ('typing”) for nine blocks, with each block lasting 30 s. Thirty-second rest periods were interleaved between typing periods (8 blocks of rest periods). ***b***, Typing speed across training blocks. Block performance was summarized as the median typing speed over the 30 s typing period. Each line represents one participant’s performance across nine blocks. Error bars represent standard error of the mean. For visualization, performance of block 1 was subtracted from the remaining blocks. Raw typing speed across training blocks without baseline subtraction is shown in the gray inset. ***c***, Learning slope per participant. Each point shows a participant’s learning slope, calculated as the sum of the group-level effect of block number and their participant-specific random slope from the linear mixed-effects model. These slopes reflect the rate of improvement in typing speed (correct keypresses per block; ***p < 0.001). Error bars represent standard error of the mean.

Participants performed an explicit motor sequence learning (MSL) task (Fig. 1*a*; Karni et al., 1995) with their nondominant hand, where they repetitively typed a 5-item numerical sequence (1-4-2-3-1) displayed on a screen as quickly and as accurately as possible. Keypress 1 was performed with the little finger, keypress 2 with the ring finger, keypress 3 with the middle finger, and keypress 4 with the index finger. Individual keypress times and identities were recorded for behavioral data analysis. Participants performed the MSL task for nine consecutive typing blocks, with each block lasting 30 s. Thirty-second rest periods were interleaved between typing blocks (eight blocks of rest periods in total). This alternating 30 s typing/rest structure was based on widely adopted paradigms in motor learning research, particularly in studies examining offline memory consolidation and sleep (Karni et al., 1995; Walker et al., 2002, 2003; Kuriyama et al., 2004).

During the typing periods of the MSL task, participants were instructed to fixate on the five-item sequence continuously displayed on the screen. To facilitate sequential training, a hexagon was shown below the to-be-pressed item and advanced to the next item only upon a correct keypress. During the rest periods, the sequence was replaced with a countdown proceeding from 30 to 0 in 1 s increments, and participants were instructed to fixate on this countdown. The experiment was designed and delivered using MATLAB (MathWorks) and Psychophysics Toolbox v.3 (Kleiner et al., 2007). Before the task began, a practice session was administered using the simplified sequence 4-3-2-1-4. For an exploratory manipulation, eight participants were randomly assigned to a “sound” condition in which keypresses 1-2-3-4 were paired with tones C-D-E-F, respectively, while the remaining 12 participants completed the task without sound. Both left- and right-handed participants were included in the sample. As these factors did not significantly influence learning rates (see Results), all participants were pooled for subsequent analyses.

#### Control (CTL) resting state task

To assess whether ripple increases during intertyping rest periods reflected learning-related processes, rather than a general feature of resting states, a subset of participants (*n* = 17; all participants except for participants 1–3) completed a post-training control resting-state task that did not involve motor learning. In this task, participants were instructed to fixate on a centrally displayed mid-gray cross that intermittently changed to either a lighter or darker gray. The control task consisted of five 60 s blocks. In each block, participants were instructed to covertly count the number of times the cross turned dark gray within each 60 s block. At the end of each block, participants verbally reported the total number of dark gray transitions and received accuracy feedback. This task served as a post-learning resting baseline, allowing us to examine hippocampal ripple activity during a nonlearning, passive attentional state.

### Motor sequence learning metric

Motor sequence performance was quantified by modeling response speed at the level of individual keypresses, filtered by sequence-level correctness (similar to Jacobacci et al., 2020). For each keypress, response time (RT) was defined as the time elapsed since the preceding keypress. To ensure data quality, RTs were preprocessed to exclude outlier responses, defined as RTs shorter than 50 ms or exceeding ±3 standard deviations from the block-wise average, thus removing likely accidental presses and attentional lapses. A keypress was considered correct if it occurred within a valid 5-item sequence pattern (1-4-2-3-1, 4-2-3-1-1, 2-3-1-1-4, 3-1-1-4-2, 1-1-4-2-3). We implemented a sliding window procedure across the continuous keypress stream to detect exact matches to these valid sequences, allowing correct sequences to be identified even after an error or mid-sequence restart. Only keypresses embedded within such correctly executed sequences were included in subsequent analyses. Typing speed was then defined as the reciprocal of RT (i.e., 1/RT) for each correct keypress, yielding a fine-grained, per-keypress measure of skilled performance. This approach ensured that performance metrics reflected both the speed and correctness of individual keypress, while accounting for the structured nature of the task.

To summarize performance at the block level, we computed the median typing speed across all correct keypresses within each 30 s typing block. The median, rather than the mean, was chosen to mitigate the influence of residual outliers, particularly given the suboptimal experimental settings in the clinical environment (e.g., participants sitting upright in bed with a laptop on a bedside table). Occasional extreme values, potentially due to momentary lapses in attention, environmental distractions, or motor interruptions, could disproportionately skew the mean. The block-level median, as a more robust measure of central tendency, reduces the impact of such outliers while still capturing overall learning trends.

### Recording system and electrode contacts localization

iEEG recordings were obtained using Behnke–Fried depth electrodes (Ad-Tech Medical Instrument) connected to an ATLAS recording system (Neuralynx), with signals sampled at 2,048 Hz. Electrode implantation was guided by clinical criteria, and only patients with electrodes targeting the hippocampus were included in the present study. Raw signals were first downsampled to 1,024 Hz and then notch-filtered at 50 Hz and its harmonics (100, 150 Hz) to remove line noise. Within each subject, contacts located within the hippocampal formation were identified via visual inspection of postoperative T1-weighted anatomical MRI scans. Bipolar referencing was then performed using the most medial hippocampal contact and its immediate neighbor (i.e., the second-most medial contact) on each hippocampal probe. For visualization, electrode coordinates were transformed to MNI space using the brainstorm-toolbox (Tadel et al., 2011; Fig. 1*a*; Supplemental Table S1).

### Artifact detection and rejection

To mitigate any impact of interictal epileptiform discharges (IEDs) and other artifacts on our results, an automated artifact detection procedure was applied to each bipolar channel prior to ripple detection, followed by visual confirmation. Artifactual time points were defined as those where any of the following metrics exceeded 4 interquartile ranges (IQRs) above the median across all time points: (1) absolute amplitudes of the raw signal, (2) absolute amplitude of the first derivative of the raw signal (i.e., sharp amplitude gradient likely caused by IEDs), and (3) amplitude of the root mean square (RMS; 100 ms window) after high-pass filtering the signal at 250 Hz. To further exclude epileptogenic activity, two additional measures were applied. First, we assessed broadband power increases: the sum power across 30 logarithmically spaced frequencies between 1 and 60 Hz (obtained via time–frequency decomposition using 7-cycle Morlet wavelets, log-transformed and *z*-scored per frequency) was flagged if it exceeded 4 IQRs above the median (same as Kunz et al., 2024). This criterion targeted the characteristic broad-spectrum power increases associated with epileptogenic events. Second, an automatic IED detection algorithm was applied (Janca et al., 2015).

All detected artifactual segments were padded by ±1 s (Supplemental Fig. S1). Furthermore, artifact-free intervals shorter than 1 s were also marked as artifacts to ensure conservative rejection. These thresholds were based on prior work investigating human hippocampal ripples (Staresina et al., 2015; Ngo et al., 2020; Kunz et al., 2024), and all automated detections were followed by visual inspection. Finally, bipolar contacts with more than two-thirds of task duration contaminated by padded artifacts were excluded. As a result, four bipolar contacts from three participants were excluded from ripple analyses (final contacts *n* = 34; participants *n* = 17). Artifact-free intervals included in the analyses are summarized in Supplemental Table S1. To rule out the possibility that variability in hippocampal pathology influenced behavioral performance, we examined whether the amount of artifact-free task time predicted behavioral outcomes. No significant relationships were observed at either the participant or block level (see Supplemental Materials: Relationship between hippocampal pathology and behaviour).

### Ripple detection

After the abovementioned artifact rejection steps, ripples were identified by an initial time domain detection procedure followed by a frequency domain criterion. Analytic criteria were based on prior human hippocampal ripple studies (Staresina et al., 2015; Ngo et al., 2020; Chen et al., 2021; Kunz et al., 2024). Signals from hippocampal contacts (i.e., continuous bipolar re-referenced time-series) were first bandpass-filtered from 80 to 120 Hz using a fourth-order FIR filter. Next, the root mean square (RMS) of the bandpassed signal was calculated and smoothed using a moving average filter with a 20 ms window. Ripples were detected based on amplitude and duration thresholds of this RMS time course. Specifically, ripple events were identified as having an RMS amplitude greater than 1.5 but not exceeding 9 standard deviations from the mean. Ripple duration was defined as the supra-threshold time of the RMS signal. Detected ripple events with a duration shorter than 38 ms (corresponding to 3 cycles at 80 Hz) or longer than 500 ms were rejected.

In addition to amplitude and duration thresholds, we further examined the spectral characteristics of each detected ripple to confirm they reflected genuine, narrowband ripple activity within the 80–120 Hz range. Following a previously established method (Chen et al., 2021), the signal was decomposed into narrow frequency bands from 1 to 200 Hz (1 Hz steps) using wavelet analysis over a 2 s window centered on the ripple. Baseline spectral amplitudes were calculated from the preripple period (−1.5 to −0.5 s) and used to normalize the ripple spectra as percentage change. Spectral peaks were identified in each ripple using MATLAB *findpeaks* function, extracting peak height, prominence, and width. Detected ripple events were rejected if they met any of the following spectral criteria: (1) the most prominent peak fell outside the ripple band (80–120 Hz); (2) high-frequency activity (30–200 Hz) outside the ripple band exceeded 80% of the ripple peak amplitude; (3) multiple peaks were present within the upper frequency range (120–200 Hz), suggesting contamination by high-frequency noise; and (4) ripple peak spectral width exceeded 3 standard deviations above the mean width for that hippocampal contact, reflecting atypically broad spectral profiles inconsistent with narrowband ripples. On average, 23.4% (SD = 9.9%) of candidate ripples during the experiment were rejected by spectral criteria. For identified ripples, we summarized the following four attributes: (1) ripple rate (Hz), calculated by using the number of detected ripple events divided by artifact-free experiment time in seconds, (2) ripple duration (ms), (3) ripple max amplitude (μV) calculated using the ripple bandpassed signal, and (4) ripple frequency (Hz) reflecting the number of oscillatory cycles in the bandpass-filtered signal per second within each ripple event.

### Statistical analysis

All statistical analyses were conducted in R using linear mixed-effects models (LMEs) via the *nlme* package. LMEs were used to account for both fixed effects of interest and random effects reflecting individual differences and repeated measures within participants and contacts. Models were estimated using Restricted Maximum Likelihood (REML), with a symmetric positive-definite covariance structure specified for the random effects.

#### Typing speed improvements across training blocks

To examine the improvements in typing speed across learning blocks, typing speed was modeled as a function of block number, with participant-specific random intercepts and slopes to account for individual differences in baseline performance and learning rates across blocks (see Model S1 in the Supplemental Materials).

#### Ripple attributes across MSL typing/rest periods and CTL task

To test whether ripple activity during intertyping rest periods reflects learning-related neural processes rather than general resting-state activity, we compared ripple attributes across three conditions: MSL typing periods, intertyping rest periods, and the post-experiment CTL task. Ripple rate, duration, amplitude, and frequency was each modeled as a function of task conditions (MSL typing, MSL rest, and CTL), with participant and contact (nested within participant) included as random factors to account for repeated measures and inter-individual variability (Model S2 in the Supplemental Materials). Each attribute was analyzed separately, and *p* values of the main effects were Bonferroni corrected across the four comparisons. For each ripple attribute showing a significant main effect of condition, post hoc pairwise comparisons were conducted using estimated marginal means with Bonferroni’s correction to compare MSL typing versus MSL rest, MSL rest versus CTL, and MSL typing versus CTL. Note that ripple attributes were extracted from identified events and averaged within each condition prior to statistical analysis.

#### Link between offline ripples and learning behavior

We first examined whether ripple rates tracked motor learning across blocks. Ripple rate was modeled as a function of block number, MSL condition (typing vs rest), and their interaction. Random intercepts were included for participants and for contacts nested within participants, to account for individual variability (see Model S3 in the Supplemental Materials). Significant interactions were followed up using estimated marginal means (*emmeans* package), focusing on condition-specific slopes of ripple rate across blocks. We tested whether these slopes significantly differed from zero.

Building on this, we then investigated whether changes in ripple rate were associated with behavioral improvements, both across participants and across blocks. To account for individual baseline differences, rest ripple rates were normalized using the formula: (rest ripple rates − typing ripple rates) / (rest ripple rates + typing ripple rates).

##### Block-level analysis

We assessed whether rest ripple rate predicted typing speed in the subsequent block. Next-block typing speed was modeled as a function of the normalized rest ripple rate from the preceding rest period. Typing speed in the previous block was included as a covariate to account for baseline performance, under the assumption that better prior performance may constrain the scope for further improvement. The LME included random intercepts and slopes for block nested within participants to reflect the hierarchical data structure (Model S4 in the Supplemental Materials).

##### Participant-level analysis

We estimated the slope of rest ripple rate and typing speed across blocks using separate LMEs (Models S5 and S6, respectively). Both models included participant-specific random intercepts and slopes for block and additionally contact-level random intercepts nested within participants for ripple rate. From each model, we extracted participant-specific random slopes, capturing individual trajectories of behavioral improvement and ripple rate change. We then computed both Pearson’s *r* and Spearman’s rank correlation between the two sets of slopes to assess whether greater behavioral gains were associated with greater increases in rest ripple rate.

## Results

### Typing speed improvements across training blocks

To test whether participants’ typing performance reliably improved across training, we examined the effect of block number on typing speed using an LME model (Supplemental Model S1). Typing speed increased significantly across blocks (*F*_(1,159)_ = 89.58; *p* < 0.001), with an increase of 0.09 correct keypresses per block (Fig. 1*b*; *β* = 0.087, SE = 0.009, *t* = 9.465, *p* < 0.001). Figure 1*c* illustrates the learning slope for each participant, derived from the fixed effect of block number (group-level slope) combined with individual random slopes, showing that all participants exhibited positive trajectories of typing speed.

To ensure robustness, we also tested whether learning trajectories differed by sound condition or handedness. There were no significant differences in learning rates between participants in the “sound” versus “no sound” conditions (*F*_(1,158)_ = 1.104, *p* = 0.295), nor between left- and right-handed participants (*F*_(1,158)_ = 0.509, *p* = 0.477). Therefore, all participants were pooled for all analyses.

### Ripple attributes across MSL typing, MSL rest periods, and CTL task

**Figure 2.**
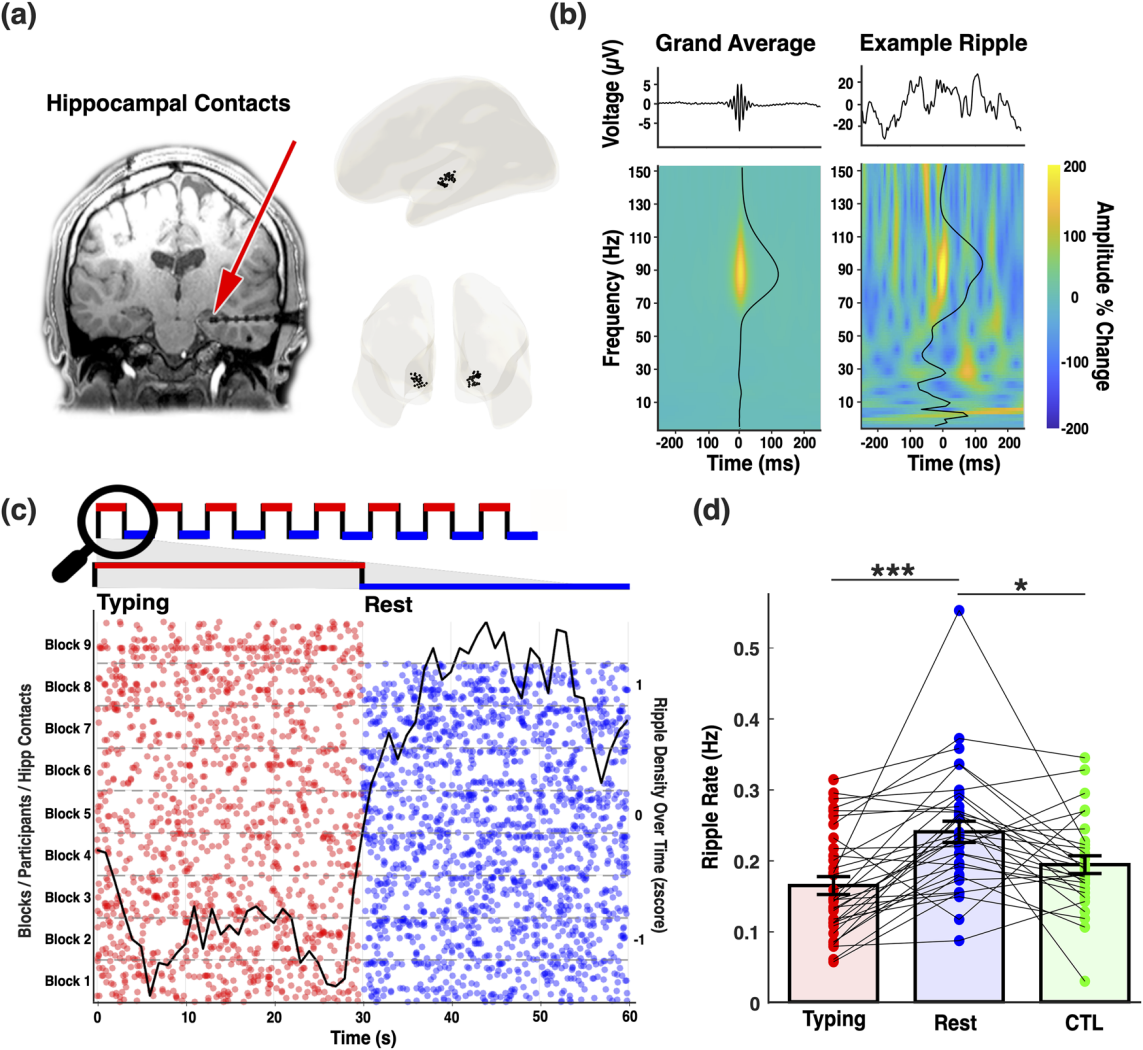
Hippocampal ripple rates are modulated by task condition. ***a***, Hippocampal contacts. Left panel, Anatomical location of an example electrode with two contacts in the hippocampus. Right panel, Hippocampal contacts from all participants rendered on a brain template in MNI space. ***b***, Hippocampal ripples. Left, Grand average of ripple-locked raw voltage trace and time–frequency decomposition from all ripples detected during the experiment across all hippocampal contacts (*n* = 34). Right, Ripple-locked raw voltage trace and time–frequency decomposition of an example ripple. Color of the time–frequency plot represents amplitude change compared with pre-event baseline (−1.5 to −0.5 from ripple center). Black line on the time–frequency plot represents amplitude density across frequency bands. ***c***, Raster plots of ripple occurrences during MSL typing and rest periods, illustrating the ripple rate increase during rest periods. Each row represents one block of typing and rest periods from each participant/contact. The overlaid black line shows the average ripple density across all MSL typing and rest blocks, concatenated into a 60 s window (0–30 s = typing; 30–60 s = rest), smoothed using a 5 s moving average, and downsampled to 1 Hz for visualization. ***d***, Bar graph of averaged ripple rate during motor sequence learning (MSL) typing and rest periods, and the control (CTL) task. Ripple rates during intertyping rest periods were significantly higher than both MSL typing periods (*p* < 0.0001, Bonferroni corrected) and the CTL task (*p* = 0.0080, Bonferroni corrected), whereas ripple rates during MSL typing and the CTL task did not differ significantly (*p* = 0.181). These findings suggest that the increase in ripple rate during intertyping rest is not simply a characteristic of resting hippocampal activity but likely reflects motor learning-related processes. Each point represents the average ripple rate for each bipolar referenced contact (***p < 0.0001; *p < 0.01). Error bars represent standard error of the mean. See Supplemental Figure S2 for a temporal analysis showing that ripple–learning relationships are distributed across the rest period rather than limited to specific boundaries.

We examined four ripple attributes (rate, duration, amplitude, and frequency) during MSL typing and rest periods (Bonferroni corrected for multiple comparisons). As shown in Figure 2*b*, the detected ripples exhibited elevated power in the 80–100 Hz range, consistent with canonical human hippocampal ripples (Bragin et al., 1999; Axmacher et al., 2008; Staresina et al., 2015; Norman et al., 2019; Vaz et al., 2019; Ngo et al., 2020; Chen et al., 2021; Kunz et al., 2024). A raster plot of ripple events pooled across all contacts and participants (Fig. 2*c*) revealed more prominent ripple activity during rest compared with typing periods. LME models examining ripple attributes (Supplemental Model S2), with task condition (MSL typing, MSL rest, and CTL task) as a fixed effect and participant and contact as random effects, revealed a significant main effect of task on ripple rate (*F*_(1,60)_ = 14.848, *p* < 0.001). Ripple rates were, on average, 0.076 Hz higher during MSL rest compared with typing periods (Fig. 2*d*; *β* = 0.076, SE = 0.014, *t* = 5.405, *p* < 0.001) and CTL task (Fig. 2*d*; *β* = 0.047, SE = 0.015, *t* = 3.132, *p* = 0.008). Ripple duration also showed a trend-level main effect of task (*F*_(1,60)_ = 3.186, *p* = 0.048, uncorrected), with longer durations during rest compared with typing periods by 1.336 ms (*β* = 1.336, SE = 0.640, *t* = 2.087, *p* = 0.123) and the CTL task by 1.526 ms (*β* = 1.526, SE = 0.685, *t* = 2.227, *p* = 0.089). However, this effect did not survive Bonferroni’s correction. No statistical differences were observed in ripple amplitude (*F*_(1,60)_ = 1.485; *p* = 0.235) or peak frequency (*F*_(1,60)_ = 1.551; *p* = 0.220).

### Link between offline ripples and learning behavior

**Figure 3.**
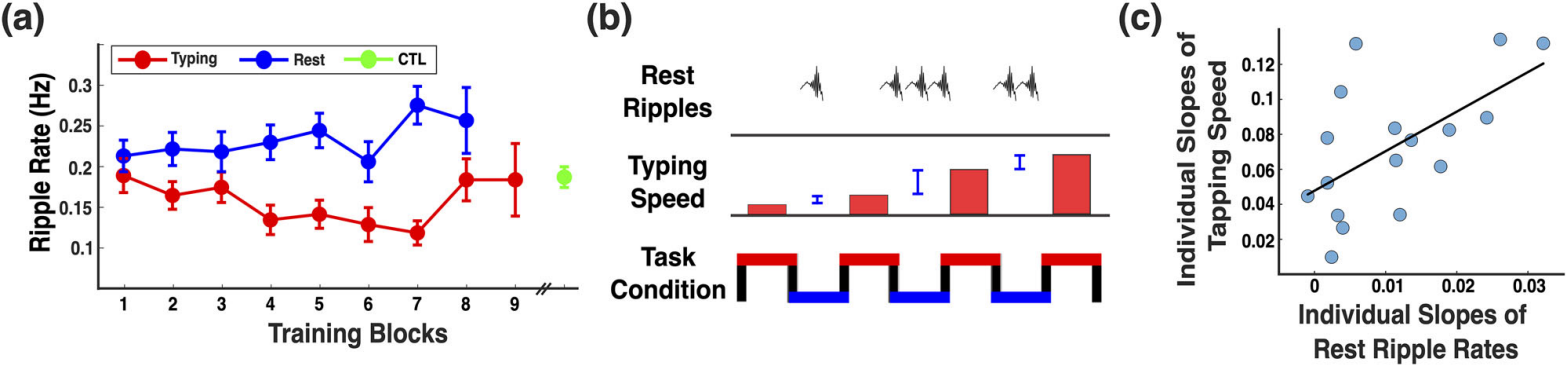
Link between ripples and learning behavior. ***a***, Ripple rate as a function of MSL blocks and task condition (typing vs rest). As block number increased, the ripple rate difference between rest and typing periods became more pronounced, suggesting learning-related modulation of ripple activity. CTL ripple rates, collected after the MSL task, are plotted at the far right for reference. Error bars represent standard error of the mean. ***b***, Schematic illustration of the finding that rest ripple rate predicts block-to-block improvements in typing speed. Bottom, Experimental task structure, with typing blocks (red) interleaved with rest periods (blue). Middle, Typing speed represented by red bars and block-to-block improvement in typing speed are indicated by blue vertical lines. Top, Ripple rates during rest blocks track block-to-block improvements in typing speed. ***c***, Individual learning slopes (typing speed across blocks) correlate with rest ripple rate slopes across participants (Spearman’s rho = 0.556; *p* = 0.022; Pearson’s *r* = 0.587; *p* = 0.013).

Is the increase in hippocampal ripples during offline periods linked to motor sequence learning? If so, ripple rates during rest periods should mirror behavioral performance increases across blocks (Fig. 1*b*). Examining MSL typing and rest ripple rates across blocks, we indeed observed a steady increase in rest ripple rates (Fig. 3*a*).

An LME model (Supplemental Model S3) revealed a significant interaction between task condition and block number on ripple rate (*F*_(1,507)_ = 6.543; *p* = 0.011), reflecting an average increase of 0.01 Hz per block in the difference between rest and typing ripple rates (*β* = 0.011, SE = 0.004, *t* = 2.558, *p* = 0.011). Post hoc tests showed a significant positive slope of ripple rate across blocks during rest periods (*t* = 2.103; *p* = 0.036), indicating an increase in hippocampal ripple rate across interblock rest periods. In contrast, the slope in the typing condition did not differ from zero (*t* = −1.514; *p* = 0.131). Together, these results suggest that ripple rate increased across blocks specifically during offline rest periods, in parallel with behavioral performance.

Next, we directly tested the association between offline ripple rates and performance increases across blocks (Fig. 3*b*). In other words, are stronger performance improvements from block *n* to block *n* + 1 accompanied by greater ripple rates in the intermittent rest period? To tackle this question, we modeled next-block typing speed as a function of normalized rest ripple rate from the preceding rest period, while including prior typing speed as a covariate to control for baseline performance (Supplemental Model S4). This model included participant-level random intercepts and slopes for block. Results revealed a significant effect of rest ripple rate on next-block performance (*F*_(1,234)_ = 12.250; *p* < 0.001; *β* = 0.058, SE = 0.026, *t* = 2.208, *p* = 0.028), indicating that greater ripple rate during rest was predictive of improved motor performance in the subsequent block.

Lastly, we directly tested the association between offline ripple rates and performance increases across participants (Fig. 3*c*), i.e., does a participant who learns faster also show a greater increase in ripple rates during rest blocks? To do this, we estimated participant-specific slopes for normalized rest ripple rate and typing speed using separate LME models (Supplemental Models S5, S6, which included random intercepts and slopes for block per participant, plus nested random intercepts for contacts in the ripple model. We then extracted the slope term for each participant and correlated the normalized rest ripple slopes with typing speed slopes. This analysis revealed a positive correlation across participants (Fig. 3*c*; Spearman’s rho = 0.556; *p* = 0.022; Pearson’s *r* = 0.587; *p* = 0.013). Participants who had higher rest versus typing ripple rate increases across blocks also showed a greater typing speed improvement across training. Importantly, all results remained unchanged when including handedness as a fixed-effect regressor, indicating that the observed ripple–behavior relationship is not driven by handedness.

## Discussion

Hippocampal ripples have recently emerged as a viable mechanism to support episodic memory processes in humans. However, beyond episodic memory, we have the remarkable ability to continuously acquire complex motor skills, from riding a bicycle to playing a musical instrument. Before becoming effortless and automatic, this kind of learning involves integrating separate movements into a coordinated sequence of actions governed by task-specific rules. Here, we asked whether the hippocampus might be involved in motor sequence learning (MSL; Fig. 1*a*). We first confirmed that all participants improved performance across experimental blocks (Fig. 1*b*,*c*). Next, we demonstrated that hippocampal ripple rates (Fig. 2*a,b*) were strongly modulated by MSL rest, MSL typing, versus CTL condition (Fig. 2*c*,*d*). Finally, we found significant associations between these offline ripple rates and motor learning. Specifically, rest (vs typing) ripple rates not only increased with block number (Fig. 3*a*) but also predicted performance changes across blocks (Fig. 3*b*) as well as across participants (Fig. 3*c*). These findings suggest that hippocampal ripples during rest periods contribute to motor sequence learning.

These results align with emerging evidence that hippocampal ripples are not exclusive to episodic memory. For example, a recent human iEEG study examined hippocampal ripples during various cognitive tasks and found that ripples were not unique to episodic memory behavior (Chen et al., 2021). Instead, ripples occurred with similar attributes during other visual perceptual tasks. Notably, ripples showed highest occurrence rates and longest durations during rest states (6 min eyes open/closed) compared with active tasks, highlighting the preferential engagement of ripples during offline states. These results dovetail with evidence that the hippocampus contributes to learning during offline periods even in tasks that show no apparent hippocampal involvement during active acquisition (Sawangjit et al., 2018; Schapiro et al., 2019). Notably, recent work in humans has also implicated hippocampal ripples during the “online” parts (encoding and retrieval) of episodic memory tasks, albeit without assessing ripples during offline delay periods in the same experiment. Several studies have replicated the link between memory retrieval and ripples, with ripples reliably increasing during episodic recall (Norman et al., 2019; Vaz et al., 2019; Kunz et al., 2024; Mishra et al., 2025; see Reithler et al., 2025 for a recent review). Fewer studies have examined ripple dynamics during the learning/encoding phases (Henin et al., 2021; Sakon et al., 2024). To address this important distinction between online and offline ripple functions, our supplementary analyses further confirmed that ripple rates during typing periods were generally low and unrelated to behavioral performance of motor learning (Supplemental Materials: Ripple Rates During Online Typing Periods). Taken together, these findings support the idea that hippocampal ripples may contribute to the encoding and retrieval of episodic memories but also might play a more general role in post-learning consolidation, even in tasks that do not require hippocampal engagement during the initial learning phase. Our current results extend prior work by suggesting that, at least in motor skill acquisition, offline ripples might play a more important role for behavioral improvements than online ripples.

How might hippocampal ripples during offline periods contribute to motor skill learning? A recent model proposes that the hippocampus may act as a sequence generator, connecting encoded items of different modalities in space/time via ripples (Buzsáki and Tingley, 2018). We speculate that during MSL rest periods, ripple events may mark moments of hippocampal reactivation of the motor sequence, leading to behavioral improvements in task performance. The relevance of offline periods for motor skill acquisition is well established, as distributed practice with frequent rest breaks enhances learning compared with massed practice with continuous training (Lee and Genovese, 1988). In light of our current findings, one tentative interpretation of this pattern is that an increased number of offline periods provides more opportunity for the hippocampus to drive reactivation and set consolidation processes in motion. Indeed, recent human fMRI work has provided evidence of hippocampal activation during awake rest after motor skill learning and its relation to consolidation (King et al., 2022). Furthermore, recent studies employing fine-grained block-by-block analyses of MSL tasks in healthy participants have shown that rest periods interspersed between typing promote consolidation at much shorter timescales (i.e., 10–30 s) than previously thought (“micro-online and -offline gains”; Jacobacci et al., 2020; Buch et al., 2021). Accordingly, the ripple engagement we observed during MSL rest periods may reflect a form of rapid consolidation via reactivation that contributes to behavioral improvements across training. However, there is ongoing debate about whether these micro-offline gains reflect genuine learning or are confounded by factors such as fatigue and recovery (see Gupta and Rickard, 2022, 2024; Das et al., 2024 for recent discussions). This issue is particularly salient in our epilepsy patient cohort, where environmental and physiological variability is inherently high. We therefore measure motor skill acquisition at a broader block-wise scale, focusing on typing speed and learning slopes to reduce sensitivity to short-term fluctuations in alertness or fatigue. This approach provides a robust measure of skill acquisition under our constraints, though future work with paradigms tailored to within-block learning dynamics in patient populations will be critical for determining the fine-grained hippocampal contributions to motor skill consolidation.

If ripple-driven reactivation of motor sequences underlies hippocampal contributions to skill learning, an important follow-up question is whether this mechanism is shared across memory domains. Notably, the involvement of hippocampal ripples in motor sequence learning parallels evidence from other domains, including spatial navigation and episodic memory. For instance, in the spatial domain, hippocampal ripples during offline rest are associated with the replay of place cell sequences, which supports navigational learning and planning (Foster and Wilson, 2006). While motor sequence learning also shares a temporal structure, it differs in its reliance on sensorimotor representations and procedural memory systems. Unlike spatial and episodic sequences, which involve flexible recombination and integration of elements, motor sequences often become rigidified with practice, eventually leading to automaticity. This distinction raises the question of whether hippocampal ripples serve a general sequencing function across domains or whether their role in motor learning is mechanistically unique. Future studies investigating how the hippocampus interacts with cortical and subcortical networks could refine models of hippocampal involvement in motor learning. Supporting this idea, a recent study showed that reactivation of engram neurons in primary motor cortex predicts motor learning performance in mice (Hwang et al., 2022), despite not directly examining hippocampal ripples. In humans, multivariate decoding of MEG data has revealed that spontaneous replay of motor sequence representations occurs in both hippocampal and sensorimotor regions during awake rest, and the extent of this replay predicts rapid consolidation gains (Buch et al., 2021). Intriguingly, in one patient from our current study with an iEEG contact in the premotor cortex, we observed ripple-locked decreases in beta-band power (Supplemental Materials: Motor Area Activity and Supplemental Figure S3), suggesting that hippocampal ripples may interact with motor cortical dynamics during offline periods. Notably, beta suppression during motor sequence execution was also observed at this contact, consistent with prior reports of motor beta desynchronization during motor planning and performance (Kilavik et al., 2013). Together, these findings support the notion that hippocampal ripples may contribute to learning across multiple domains by facilitating sequence reactivation but also highlight the need to clarify how this general mechanism is adapted to the specific demands of motor skill acquisition.

Several limitations and directions for future research should be considered. First, the correlational nature of our study precludes firm conclusions about causality. Future work directly manipulating ripple activity will be needed to adjudicate whether ripples per se mediate learning or whether they reflect other processes beneficial for performance improvements. Second, during the rest periods, participants were instructed to focus on a countdown timer to prevent disengagement, which may have moderately engaged cognitive processes. The observed ripples during rest periods might thus partly reflect ongoing cognition rather than purely offline memory reactivation. Future studies could address this by comparing ripples during passive rest (e.g., fixation) and cognitively engaging conditions. A further limitation concerns the specificity of the behavioral association observed. Due to constraints in experiment time and task complexity feasible in our patient cohort, we did not include a separate control condition (e.g., random or nonsequential tapping). This precludes us from conclusively attributing observed ripple–behavior associations to sequence learning specifically, as opposed to general motor performance or planning. Future studies utilizing motor control conditions will be critical for precisely delineating the contribution of hippocampal ripples to different components of skill acquisition. Lastly, the mean learning curve observed in our study appears shallower than previous MSL studies, likely due to our epilepsy cohort’s diverse clinical backgrounds and age range (Table 1). Additional factors such as baseline motor speed, cognition, and medication may also have influenced learning. Despite this variability, we still observed significant block-wise improvements, consistent with prior reports.

In conclusion, our findings integrate and extend prior work in the human and animal hippocampus to suggest that hippocampal ripples during offline periods are associated with motor skill learning, reinforcing the view that hippocampal ripples serve as an internally generated and state-dependent mechanism for learning beyond episodic memory.

## Data Availability

Derivative data, MATLAB and R scripts, and results presented in all figures will be publicly available on the Open Science Framework (https://osf.io/9r8pu/).
